# Editorial: Women in bone research

**DOI:** 10.3389/fendo.2025.1561904

**Published:** 2025-03-10

**Authors:** Monica De Mattei, Katherine A. Staines, Michaela Tencerova

**Affiliations:** ^1^ Department of Medical Sciences, University of Ferrara, Ferrara, Italy; ^2^ Centre for Lifelong Health, School of Applied Sciences, University of Brighton, Brighton, United Kingdom; ^3^ Laboratory of Molecular Physiology of Bone, Institute of Physiology of the Czech Academy of Sciences, Prague, Czechia

**Keywords:** bone research, women in science, clinical studies, animal models of bone diseases, sex differences

This Research Topic entitled “*Women in Bone Research*” showcased 15 articles by women scientists, highlighting novel findings in basic and clinical musculoskeletal research.

Among the basic bone research studies, Gilbert et al. used human mesenchymal stem cells (Y201) to develop a 3D *in vitro* model of osteocytes, which highlighted numerous genes implicated in osteoarthritic pain, as well as inflammation and bone remodeling. The findings contribute to a deeper understanding of osteoarthritis pathology and may guide the development of targeted therapies through use of this model. Further work from this group in Jones et al. compared published data sets to reveal a wide array of sex-regulated genes that are also significantly regulated by pathophysiological loading in osteocytes. Their work highlights pain related pathways which may underpin elevated pain susceptibility in females with osteoarthritis and offers potential therapeutic targets. A final study on osteocytes by Yee et al., examined the skeletal effects of glucocorticoids and PTH(1-34), alone and combined on perilacunar canalicular remodeling (PLR). They found sex-dependent differences in responsiveness to glucocorticoids and PTH(1-34) and ultimately no evidence that PTH(1-34) could offset glucocorticoid-dependent effects on PLR, thus highlighting that further studies are required.

Verlinden et al. analyzed the bone phenotype of Klotho deficient mice (kl/kl), an animal model of accelerated aging, in comparison to chronological aged mice. The major differences were in trabecular bone volume, serum calcium and phosphate levels and *Trpv6* expression, which might contribute to better understanding of the mechanism behind the accelerated aging related to the regulation of calcium metabolism. Ali et al. showed that the natural compounds Apigenin and Rutaecarpine, plant-derived antioxidants enhanced osteogenic differentiation of human bone marrow stromal stem cells (hBMSCs) derived from elderly females. Further, these molecules reduced oxidative stress and the accumulation of senescence cells, suggesting the therapeutic potential of these natural compounds in the age-related bone loss. Prabhakaran et al. explored a novel approach to demonstrate that by guiding the fusion of differentiated rat osteoblast cell spheroids, it is possible to engineer bone macro-tissues that mimic physiological osteogenesis both morphologically and molecularly. This technique holds promise for advancing tissue engineering and regenerative medicine, particularly for bone repair and replacement.

An RNA-seq study by Bourne et al., showed divergent chondrocyte phenotypes in *ex vivo* hip cap articular cartilage and metatarsal growth cartilage cultures. They also revealed that hydrostatic pressure application downregulated biological processes including ossification, connective tissue development, and chondrocyte differentiation. These data therefore provide novel genetic targets for osteoarthritis research.

Finally, Tian et al. summarizes findings from preclinical studies on lactoferrin, a multifunctional protein, which has emerged as a promising therapeutic opportunity for bone diseases. The authors provide a critical discussion on the opportunity to use lactoferrin-derived peptides as potential therapeutic agents for the treatment of orthopedic and metabolic bone diseases and highlight the need to develop strategies for the delivery of lactoferrin or derived peptides to bone.

Several papers of the Research Topic focused on different clinical aspects with the aim to identify risk factors for bone health and potential therapies. A study by Harada et al. found that fat content in vertebral bone marrow (BM) and muscle is associated with increased bone fractions and metabolic complications. Notably, a history of gestational diabetes was significantly associated with a higher proton density fat fraction (PDFF) of the vertebral BM, independent of age and BMI, thus highlighting vertebral BM PDFF as a potential biomarker for the assessment of bone health in premenopausal women as a risk factor of diabetes. An observational study by He et al. investigated the association between the atherogenic index of plasma (AIP) and BMD among adult women using National Health and Nutrition Examination Survey (NHANES). They found a negative correlation between the AIP and total BMD measured by DXA, thus suggesting that high AIP might serve as a good biomarker for a low BMD and contribute to the prevention of the osteoporosis. Aparicio-Bautista et al. analyzed the association between 3 single nucleotide variants MARK3 (rs11623869), PLCB4 (rs6086746) and GEMIN2 (rs2277458), with BMD and vitamin D levels in Mexican women to create genetic risk score (GRS). GRS revealed significant associations between the variants and hip and femur neck BMD and vitamin D. Thus, these findings may contribute to early detection of the pathogenesis of osteoporosis. In the systematic clinical review Liu et al. investigated the efficacy of 5 different Chinese fitness exercises (Baduanjin, Taijiquan, Wuqinxi, Yijinjing, and Liuzijue) both alone and in combination with drug therapy in the treatment of decreased BMD in postmenopausal women. The outcome of the studies confirmed the positive effect of the Chinese fitness trainings, even more effective with drug treatments. However, the small number of studies and participants limits definitive conclusions; further clinical research with larger cohorts is needed.

Chen et al. investigated the causal relationship between hormonal and reproductive factors and low back pain (LBP), by using Mendelian randomization (MR) analysis. Their results showed that early menarche, first birth, last birth, and first sexual intercourse may reduce the risk of LBP. This confirms that reproductive hormones, particularly estrogen, may play a protective role against LBP, possibly by influencing intervertebral disc metabolism and bone health. The paper by Ren et al. analyzed the clinical characteristics and prognostic factors of survival for patients with bone metastases of unknown origin, in a large population-based study including 1224 cases. They found these patients have extremely low 1-year survival rates, only 14.5% for overall survival and 15.9% for cancer-specific survival. In addition, they revealed that radiotherapy and chemotherapy were significantly correlated with prognosis, suggesting that these treatments may be effective in prolonging survival and the need for further research to evaluate the efficacy of treatments. A final clinical aspect explored in this Research Topic was delayed bone healing, reported in the case report by Ryskalin et al. The authors conducted a critical analysis and summary of the present information deriving from basic research and clinical reports concerning the beneficial effects of extracorporeal shockwave therapy (ESWT) on bone healing. Further they showed the promising outcomes of a delayed ulnar fracture treated with focused high-energy ESWT, suggesting EWST as a safe and promising alternative to surgery in the treatment of delayed union and nonunions.

In conclusion, this Research Topic highlights the wide-ranging contributions of women researchers, covering the topics from fundamental scientific investigations to practical clinical applications. Overall, it provides a comprehensive overview of current research in bone biology, and research directions, with particular attention to aging and sex differences, highlighting the complexity of bone homeostasis and the potential for novel therapeutic interventions. By amplifying the voices and relevant research of women scientists, this Research Topic may inspire future generations of women to pursue careers in bone biology and contribute to the advancement of this crucial field.

